# The CAP cancer protocols – a case study of caCORE based data standards implementation to integrate with the Cancer Biomedical Informatics Grid

**DOI:** 10.1186/1472-6947-6-25

**Published:** 2006-06-20

**Authors:** Jonathan Tobias, Ram Chilukuri, George A Komatsoulis, Sambit Mohanty, Nicholas Sioutos, Denise B Warzel, Lawrence W Wright, Rebecca S Crowley

**Affiliations:** 1 Department of Pathology, University of California at Los Angeles School of Medicine, Los Angeles, CA, USA; 2 SemanticBits, LLC, Reston, VA, USA; 3 National Cancer Institute Center for Bioinformatics, National Institutes of Health, United States Department of Health and Human Services, Rockville, MD, USA; 4 Department of Pathology, University of Pittsburgh School of Medicine, Pittsburgh, PA, USA; 5 Lockheed Martin Information Technology, Rockville, MD, USA; 6 Office of Communications, National Cancer Institute, Rockville, MD, USA; 7 University of Pittsburgh Cancer Institute, Pittsburgh, PA, USA; 8 Center for Biomedical Informatics, University of Pittsburgh School of Medicine, Pittsburgh, PA, USA

## Abstract

**Background:**

The Cancer Biomedical Informatics Grid (caBIG™) is a network of individuals and institutions, creating a world wide web of cancer research. An important aspect of this informatics effort is the development of consistent practices for data standards development, using a multi-tier approach that facilitates semantic interoperability of systems. The semantic tiers include (1) information models, (2) common data elements, and (3) controlled terminologies and ontologies. The College of American Pathologists (CAP) cancer protocols and checklists are an important reporting standard in pathology, for which no complete electronic data standard is currently available.

**Methods:**

In this manuscript, we provide a case study of Cancer Common Ontologic Representation Environment (caCORE) data standard implementation of the CAP cancer protocols and checklists model – an existing and complex paper based standard. We illustrate the basic principles, goals and methodology for developing caBIG™ models.

**Results:**

Using this example, we describe the process required to develop the model, the technologies and data standards on which the process and models are based, and the results of the modeling effort. We address difficulties we encountered and modifications to caCORE that will address these problems. In addition, we describe four ongoing development projects that will use the emerging CAP data standards to achieve integration of tissue banking and laboratory information systems.

**Conclusion:**

The CAP cancer checklists can be used as the basis for an electronic data standard in pathology using the caBIG™ semantic modeling methodology.

## Background

### The Cancer Biomedical Informatics Grid

The Cancer Biomedical Informatics Grid (caBIG™) [1,2] is a voluntary association or Grid dedicated to creating an interoperable network of data and analytical services that benefits the cancer research community. Currently, there are over 800 participants in caBIG™ from a variety of institutions including National Cancer Institute (NCI) funded cancer centers, universities, government, the commercial sector and patient advocacy groups. The program is funded by the NCI.

The NCI chose to conduct the three-year caBIG™ pilot to determine how to integrate multiple heterogeneous data sets and analytical resources to help answer complex biomedical questions. Traditionally, development of biomedical data systems that support integration of multiple data types has required the creation of large, complex, highly centralized information systems. Such systems tend to be fragile, expensive, and inflexible in response to new data or analytical methods that are developed within the biomedical research community. Thus, caBIG™ will help create the technology and the community that are required to allow locally developed data systems to interoperate in novel ways that were not necessarily anticipated by their developers.

There are two distinct problems faced in creating interoperable systems. First, the systems must be capable of exchanging information ('syntactic interoperability'). Second, the systems must be able to utilize the information that has been exchanged ('semantic interoperability'). The guiding principles for achieving both syntactic and semantic interoperability have been described previously [3].

### caBIG™ – a biomedical informatics community

Most activities in caBIG™ are carried out through the framework of *workspaces *dedicated to content areas. A *domain workspace *is a group of people that are focused on a particular area of biomedical research. Workspaces meet on a regular basis by web- or tele-conference and face-to-face on a quarterly basis. These meetings serve to keep members up to date on current project development within caBIG™, encourage the development of data standards and help to create a climate conducive to data sharing and cooperation. Currently, caBIG™ has four domain workspaces: Clinical Trial Management Systems (CTMS), Tissue Banks and Pathology Tools (TBPT), *In Vivo *Imaging (IVI) and Integrative Cancer Research (ICR).

In addition to the domain workspaces, caBIG™ maintains two *crosscutting workspaces*. The crosscutting workspaces create and maintain the technological and sociological structures that allow for interoperability between data systems created with a federated development model. The Architecture workspace creates the Grid infrastructure that provides for syntactic interoperability and the Vocabulary and Common Data Element (VCDE) workspace provides oversight of the semantic part of caBIG™ interoperability. As part of the activities of the two cross-cutting workspaces, the caBIG™ program is extending existing software engineering methodologies to support both syntactic and semantic interoperability. Developers in domain workspaces within caBIG™ and outside of caBIG™ may utilize these methodologies to achieve caBIG™ compatibility, and ultimately to facilitate the development of a grid of syntactically and semantically interoperable systems.

In this manuscript, we provide an in-depth example of this methodology, using a single complex domain model – the College of American Pathologists (CAP) cancer checklists. The purpose of this manuscript is to illuminate the basic knowledge and data representations that are created, and the work processes used to create them. Additionally we will describe how existing systems within and outside of caBIG™ are utilizing these representations, and how multiple systems may use them to move towards semantic interoperability.

### Rationale for a domain standard in pathology

In the past decade, several strategic research initiatives have focused on providing access to small repositories of tissue specimens and tissue related data to researchers across networks of institutions [4-9]. To date, these groups have largely focused on acquisition of prospective samples, which can be collected under controlled conditions to ensure applicability of the full range of molecular techniques.

Another potential source for these specimens is the set of clinical archives maintained by surgical pathology departments across the country. These paraffin archives are well-maintained and have been shown to yield adequate and retrievable specimens (unpublished findings). Although retrospective samples are not adequate for all purposes, many research methods can be successfully applied to these specimens, including DNA-based methods and immunohistochemistry. Additionally, analysis of clinical reports associated with the paraffin archives provides a unique view of human disease across time.

To be useful to cancer researchers, both prospective and retrospective tissue samples must be annotated with the relevant pathologic and clinical information, so that researchers can query and retrieve tissues based on inclusion and exclusion criteria. These criteria include pathologic features such as diagnosis, anatomic location, and histologic subtype, as well as specific clinical features such as age, gender, clinical stage, and outcome. Capturing these key pathologic and clinical annotations for research purposes is typically a manual process of data identification from textual diagnostic pathology reports and re-entry of key features into research registries.

Until recently, opportunities for automating the process of tissue annotation have been very limited, because the relevant information is maintained as unstructured data in free-text fields of proprietary laboratory information systems (LIS), and is not available to researchers. But advances in structured reporting over the past several years have changed this landscape. The CAP cancer checklists [10] are a carefully constructed set of data elements for describing the relevant pathologic information in most human cancers. The nearly ubiquitous usage of the CAP cancer checklist data elements provides a unique opportunity for automating tissue annotation. If coded information could be harvested from clinical systems, this data could be used to automate annotation of tissue specimens. The CAP checklists are published in Adobe PDF and Microsoft Word formatted documents, and not as an electronic data standard. Although a commercial controlled vocabulary set is available [11], the absence of a true data standard has impeded sharing of this important data and individual LIS represent these checklists in idiosyncratic ways.

Thus, the development of the CAP cancer checklist models for caBIG™ was undertaken to implement a computer-based, and standard representation of an existing and accepted paper standard, in order to enable free interchange of synoptic pathology data among clinical and research information systems.

### CAP cancer checklists as a domain standard in pathology

**Figure 1:**
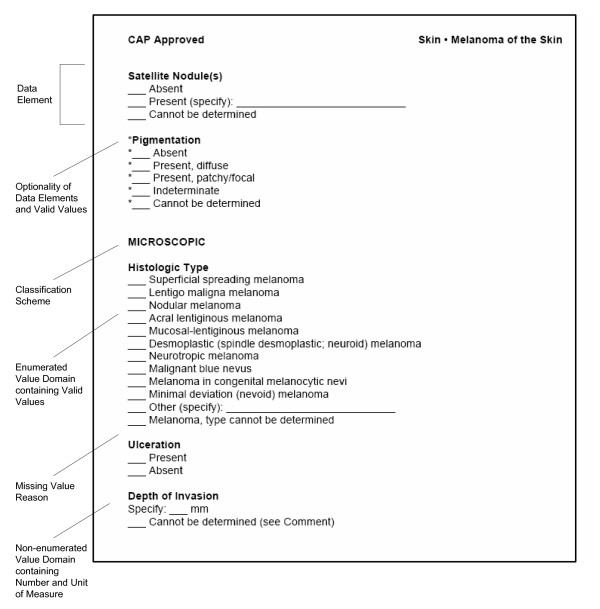
**Fragment of CAP cancer checklist for melanoma**. Fragment of CAP cancer checklist for cutaneous melanoma showing relationships to ISO/IEC Administered Components. Checklist text reproduced with permission of the College of American Pathologists.

**Figure 2:**
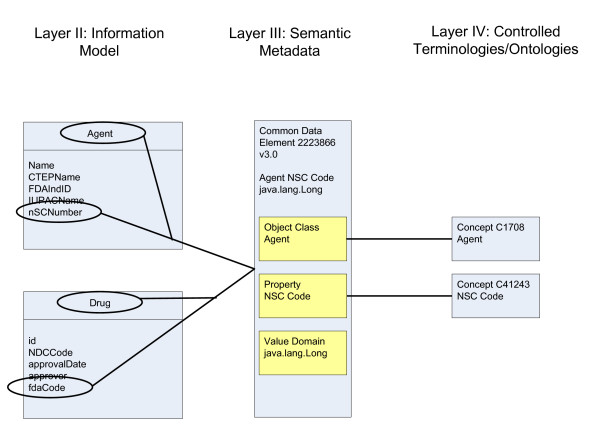
**Relationships of semantic model layers**. The threelayers of the semantic model include Layer II: information model, Layer III: semantic metadata and Layer IV: controlled terminologies. The figure illustrates two information systems using different class and attribute names, but annotating these UMLentities with the same concept. The resulting CDE will be shared between systems.

In their current format, the CAP cancer checklists consist of a series of reporting guidelines for diagnostic surgical pathology reports for 45 important human cancers [12]. Together, these cancers represent the vast majority of clinically significant neoplasms. Each guideline consists of (1) a checklist specifying the data elements of the specimen and tumor that should be included in the diagnostic pathology report, as well as the valid values that these data elements may take (Figure 1) and (2) a detailed protocol providing definitions and further information about the scientific basis for assessing these variables.

Each protocol and checklist was developed by a separate panel of subspecialty experts for that organ system, often representing differing schools of thought [10,13]. In each case, expert panels reviewed the existing literature to determine which features provided the most important data for clinical decision-making.

In addition to specifying the data elements and valid values, the CAP cancer checklists provide other useful information for creating structured metadata (Figure 1). The paper standard (1) logically groups data elements together by surgical procedures or type of examination (macroscopic vs. microscopic) to which they apply, (2) distinguishes between required data elements for which there is unequivocal scientific evidence of their value, and optional data elements which do not meet that threshold, and (3) maintains relevance with revised versions published as new data becomes available regarding prognostic factors and clinical outcomes.

The American College of Surgeons (ACS) Commission on Cancer (CoC), recognizing the importance of this standard, has mandated that all ACS CoC-approved cancer centers provide the CAP checklist items in their reports. This requirement, however, does not specify how these items should be addressed, only that they are addressed somehow. Some LIS vendors have attempted to represent the CAP models within their own relational databases in order to preserve the information as coded data. But many other vendors have chosen to simply store and present the information as free-text. A common data standard which permits interchange among clinical and research systems is urgently needed to advance tissue-based research.

The CAP cancer checklists are an example of a common conceptual model that already exists between multiple non-interoperable systems. The existence of such a conceptual model is necessary but not sufficient to achieve either syntactic or semantic interoperability. Consequently, the overarching goal of this project was to represent the meaning of the paper based CAP protocols in such a way that they would support semantic interoperation across numerous systems inside of and outside of pathology.

### The Cancer Common Ontologic Representation Environment (caCORE) – a four layer approach to interoperability

True interoperability has two components: syntactic interoperability, which concerns itself with the ability to exchange information; and semantic interoperability, which is the ability to understand and use the information once it is received. The caBIG™ program utilizes a four-layer approach to interoperability. One layer is concerned with the syntactic component of interoperability, while the remaining three layers are concerned with the semantic part of interoperability. These layers are (1) interface integration, (2) information models, (3) semantic metadata, and (4) controlled vocabularies and ontologies. When used together they provide a mechanism that allows for easy transfer of information and unambiguous interpretation of the information once it arrives. To assist developers in deploying these layers in their data system, the caBIG™ program created a set of supporting resources and tools which together are known as caCORE. Additionally, the caBIG™ program provides a set of compatibility guidelines that describe increasing levels of maturity (from Legacy, through Bronze, Silver and Gold) in these areas [3]. A system that has reached the 'Silver' level of maturity in all four areas is considered ready to connect to the caGrid [14].

#### Layer I – interface integration

The syntactic component of caBIG™ interoperability is maintained in the interface integration layer. This is achieved by requiring a well described, object oriented application programming interface (API) that is the primary mechanism by which caBIG™ users will interact with the data or analytical service. This API can be created in a computer language of the developers' choosing as long as it is object oriented. The data objects created by these information systems are moved from place to place on the caGrid by serializing them into the eXtensible Markup Language (XML) and then deserializing them back into objects when they reach their targets. This paper is meant to primarily address issues regarding semantic interoperability, so we do not discuss Layer I further. The interested reader may consult other resources for a more detailed description [15,16].

#### Layer II – information models

The base layer of the semantic interoperability step is the representation of a data system in the form of an information model in the Unified Modeling Language (UML) [17]. This model has two essential characteristics. First, it must exactly mimic the structure of the object oriented API that the system is deploying. Second, it must be a *domain information model *that represents an understanding of the scientific domain, including both the entities that are involved as well as the relationships between those entities.

The purpose of information modeling is two-fold. First, it is meant to direct the development of the information system itself. Using standard model driven architecture (MDA) methods and tools, the UML models may be used to create the database and APIs that access those tables using an intermediary object layer. Second, it is meant to describe a complex biomedical system as an interaction of the conceptual entities that interact within that system. Since all biomedical domains can be viewed as interactions between a finite set of conceptual entities, information modeling helps to increase the likelihood that two different data systems will present information on a common conceptual entity. These entities can provide the lingua franca for interoperation (see below).

#### Layer III – semantic metadata

As useful as the information model is to convey the semantic description of a data system, it is insufficient to ensure semantic interoperability. Consider an object on the caGrid, as represented by the XML below:

<Agent>

   <name>Taxol</name>

   <NSCNumber>007</NSCNumber>

</Agent>

This represents an entity called an 'Agent' with two attributes, its name is 'Taxol' and it has NSC Number '007'. Given that this is data on the caGrid, a recipient might conclude that this describes the drug Taxol and that the Nomenclature Standards Committee (NSC) of the US Food and Drug Administration gave it an ID number of '007'. However, in the absence of specific information about the meaning of the class and attribute names, another interpretation seems equally likely, namely that this describes a spy with code name 'Taxol' who has been given the number '007' by the US National Security Council (NSC).

While the example above is obviously contrived, it underscores the need for additional information about the meaning of the classes and attributes. This 'data about data' is referred to as semantic metadata and encompasses a description of the entity itself (i.e. the UML class), the characteristic of the entity being recorded (the UML attribute) and what constitutes a valid value for that attribute. Within caBIG™, such information is stored in the cancer Data Standards Repository (caDSR) an enterprise class application that implements the ISO 11179 metamodel; an international standard for describing semantic metadata.

#### Layer IV – controlled vocabularies and ontologies

Although the ISO 11179 metamodel and caDSR provide a formalism to describe arbitrary semantic metadata, it has not completely resolved the problem because (as seen above) words often have multiple meanings. In addition, words are not formally computable, i.e., a machine will not necessarily be able to determine whether two distinct natural language strings represent the same entity or attribute. The solution to this problem is the use of concept-based terminologies or ontologies with clear definitions. Specifically, we want to describe the semantic metadata in two ways: using natural language terms for human consumption, and using a series of computable codes that reference concepts in a controlled terminology. When UML classes and attributes are mapped to such codes, it is possible for a machine to determine if two distinct classes and attributes refer to the same entity and characteristic regardless of the names given to them by their developers. When instance data is mapped to such codes, it becomes possible to interoperate across repositories and perform analysis on the data itself. In this way, if a second developer created a system that used the same class and attribute names in a system for managing national security interests, they would annotate these elements with different vocabulary concepts. This would result in generation of unique common data elements (CDEs) rather than reuse of those created for the cancer domain.

#### Supporting interoperability among systems

The four layers described above provide a technical means to achieve semantic and syntactic interoperability in caBIG™. This is depicted in Figure 2. Consider two data systems, shown as the classes 'Agent' and 'Drug' on the left of the figure. The systems use different words to describe the same class, but they are both mapped to the same concept within the controlled terminology (C1708). As with the classes, the attributes have different names, but two attributes Agent.nSCNumber and Drug.fdaCode have been mapped to the same CDE (ID 2223866v3.0). This indicates that given two objects corresponding to these classes where the value of Agent.nSCNumber = Drug.fdaCode, the two objects are providing information about the same instance of the same entity.

This technology allows the identification of interaction points between data systems that were created by distinct groups working without direct interaction, which we refer to as 'Grid keys' because of their analogy to foreign keys in relational database management systems (RDBMS). Thus, using this technology it is possible to create aggregated data sets formed by the Cartesian product of two object oriented data systems, just as it is possible to create aggregated virtual tables in an RDBMS using foreign key relationships. In addition, it is possible to use these keys to 'jump' from one data system to another, allowing a richer exploration of the data space. Finally, with the appropriate Grid infrastructure, it should be possible to do a distributed query, using the results of a query into one data system as an input to a second.

## Methods

### Creating information models with the UML

Information models provide an abstract formal representation of the conceptual or physical entities in a domain. caBIG™ Information models are constructed as UML class diagrams. The UML [17] is a non-proprietary language for constructing, visualizing, and documenting the artifacts of software engineering. UML notation is software programming language neutral. It provides a standard set of diagrams which depict basic relationships within a software system. A UML class diagram is one kind of UML diagram that depicts a collection of static model elements such as physical or conceptual entities and their relationships.

In a UML class diagram specific conceptual entities are represented by *classes*. Each *class *may have *attributes *that describe specific characteristics of these entities. Classes are related to other classes by *relationships*, which are represented by arcs between classes in the UML diagram. *Associations *are "peer-to-peer" relationships between classes. The names and cardinality of the ends of the associations are marked near the box that delimits the classes. Other associative relationships include *aggregations *and *compositions*, which are used to model "whole/part" relationships between classes. *Generalization *relationships model inheritance between classes. The class that is *generalization *of a concept is referred to as the *superclass *and the class that is specialization of a generic concept is referred to as the *subclass*. Subclasses inherit attributes and methods from their superclasses. *Enumerations *are UML stereotyped classes that provide a list of named values.

We used Enterprise Architect (EA) [18] as the UML modeling tool for this project due to its low cost and high performance. During modeling, each UML class, attribute and enumeration value was annotated with a human readable definition of the semantic meaning of that class or attribute. These definitions were created as tagged values in UML, which provides a method for adding additional information to a UML stereotype. Models annotated with these definitions were then saved in the XML Metadata Interchange (XMI) [19] format for further processing.

### Semantic metadata based on the ISO/IEC 11179 standard

Semantic interoperability requires that the meaning of the data within the system or analytic services performed by the system is unambiguous. It must be interpretable by both humans and computers. In order to achieve this – a great deal must be known about the meaning and form of the data. *Semantic metadata *provides the 'data about data' needed to interpret the meaning and form of data and to determine the relationship of one datum to another. Therefore, semantic metadata must have a common, uniform structure, and must be universally available for inspection, discovery and inference.

The basic representation used for semantic metadata within caBIG™ is defined by ISO/IEC 11179 – a standard for metadata structure and registration. This specification was developed for the specific purpose of facilitating worldwide metadata standardization by providing guidance on the framework itself (Part 1), the classification of data (Part 2), the semantic structure of data (Part 3), formulation of definitions (Part 4), naming and identification (Part 5), and guidance and instruction of the registration of metadata (Part 6).[20]

*Data elements *that conform to ISO/IEC 11179 must be associated with one *data element concept (DEC) *and one *value domain*. The DEC defines the meaning of the datum. Each DEC must have one and only one *object class *that describes the real world or conceptual entity and one and only one *property *that describes some characteristic of that entity. Both object classes and properties may also take one or more *qualifiers *that modify the meaning of the object class or property. The *value domain *represents the set of *permissible values *that are valid for this datum. Value domains are annotated with representation terms that classify the data element according to the category of data stored in the data element (e.g. indicator, code, number). Value domains may be either enumerated (e.g. as a set of valid values) or non-enumerated (e.g. as a number or string). Data elements may be aggregated together as *classification scheme items *belonging to a *classification scheme*, which provides a method for grouping data elements into a logical hierarchical framework.

### The caDSR – a metadata registry

Semantic metadata of all Silver-compatible caBIG™ systems are made available through the caDSR – a conforming implementation of ISO/IEC 11179 standard for metadata registries [21]. Mechanisms for consumption of caDSR metadata include public access through web-based browsers and APIs. These tools support a variety of ways to search for, download, deploy and explore collections of caDSR metadata. The web-based tools include features that allow data elements to be organized into one or more forms. caDSR forms record usage of caDSR content, further enhancing the items' metadata. Forms and caDSR metadata can be downloaded from caDSR into a Microsoft Excel or XML format as well as accessed by APIs. An understanding of the rules and semantics of the structured metadata registered in caDSR, such as forms, ISO/IEC 11179 metamodel and UML class diagram metadata, further enhances the ability for humans and machines to unambiguously interpret and understand the associated data.

The caDSR supports metadata lifecycle management using workflow and registration status, from *Draft New *to full maturity as a registered *Standard*. The software helps to ensure consistency in naming conventions, application of common business rules, and elimination of semantically duplicate metadata, thus helping to achieve harmonization across projects and research domains.

The caDSR conforms to ISO/IEC 11179 Edition 2 Parts 1–6, and also contains NCI Center for Bioinformatics (NCICB) extensions based on NCI's goal of supporting semantic interoperability. One of the most important extensions is the linkage of caDSR-structured metadata to NCI Enterprise Vocabulary Services (EVS), in particular to the NCI Thesaurus. ISO/IEC 11179 described the use of concepts as an optional feature of object class. In caDSR, the use of concepts from controlled vocabularies is mandatory for object class, property, qualifiers and representation terms and optional for value meanings. Some values are not linked to concept codes because the instance data is unstructured text or simply not suitable as a concept in the NCI Thesaurus. Binding these ISO/IEC 11179 semantic components to concepts in the NCI Thesaurus endows a tenable layer of semantics to the already rich structure of caDSR metadata by virtue of the ontologic description (NCI Thesaurus) and mappings to synonyms in over 50 other biomedical terminologies (NCI Metathesaurus) associated with each concept.

### Generating caDSR CDEs from UML

The process of creating and registering semantic metadata as annotations of an information model in the caDSR is facilitated through the caCORE Software Development Kit (SDK) – a set of software tools and applications. The key components are Semantic Connector, Semantic Integration Workbench (SIW) and UML Loader. These tools are used by the software engineer to transform UML information models into caDSR-structured metadata. Each tool performs a specific role in the transformation process.

The UML information model exported to XMI is first processed by the Semantic Connector application. All UML element names for classes and attributes are matched to NCI Thesaurus concepts generating a tabular formatted report indicating the retrieved NCI Thesaurus concepts. Using the SIW tool, developers and NCI vocabulary experts manually revise the automated matches to produce a semantic annotation for each UML entity. Semantic Connector is applied again to produce an annotated XMI file, which contains all of the metadata derived from the original UML model in addition to the semantic information derived from the semantic annotation process. The final annotated XMI file is then used to review all associations and datatypes.

Selected components of the annotated information model are then transformed into caDSR metadata, using the UML Loader. For each potential data element that could be generated from the information model, UML Loader first determines whether a data element exists which is equivalent in semantic meaning on the basis of existing DEC concept annotations and other associated metadata. If an exact match is detected, an existing data element will be used by designation. In these cases, truly equivalent data will share an identical data element in the caDSR. New data elements are added only when they are semantically unique.

UML Loader creates caDSR concept classes, ISO/IEC 11179 classification scheme and classification scheme items from UML packages, object classes from UML classes and tagged values, and properties from UML attributes and tagged values. It preserves UML class associations and generalizations as caDSR object class relationships (an NCICB ISO/IEC 11179 extension). UML Loader also detects attribute data types and maps them to generic non-enumerated ISO/IEC 11179 value domains [15]. After the model has been loaded, model owners must use the caDSR web-based tools to create enumerated value domains and value meanings linked to EVS concepts, and associate them with the automatically generated data elements, replacing the non-enumerated value domains with the enumerated ones during *post-load curation*. For the large number of value meanings required for the CAP cancer checklists model, an existing caDSR batch process was modified to create enumerated value domains from a specially formatted Microsoft Excel spreadsheet.

### NCI Thesaurus

NCI Thesaurus was selected to provide all controlled terminology and foundational semantics for this project. NCI Thesaurus is the central reference terminology within the NCI EVS integrated suite of resources and services – designed to meet the controlled terminology needs of NCI and its partners – as well as within the caBIG™/caCORE bioinformatics architecture [22-25]. It plays a key role in the design of the project and its integration with other resources as part of the caGrid architecture.

NCI Thesaurus is a concept-based terminology system that uses description logic to enforce logical consistency and provide a formal model with computationally tractable semantics [26]. Each concept represents a single specific meaning, and includes multiple terms, codes, text definitions, and other properties that reflect that meaning. A concept can also be defined by formal description logic "role" relationships between it and other concepts, embodying the definitional criteria that logically make it a subtype of its parent concept(s) and distinguish it from sibling concepts with the same parents. Concepts are arranged in disjoint subsumption hierarchies under eighteen root nodes, such as *Activity *and *Gene*, with each step down from parent to child concept representing some added specialization of meaning.

A concept used in this project, *Acral Lentiginous Melanoma*, provides useful illustration of these features (Table 1A). This subtype of *Cutaneous Melanoma *is clearly identified by the term and text definition. The role relationships reflect much of the same, and some new, information, in a form that the description logic classifier can use; many are in fact inherited through the description logic system from concepts higher up the hierarchy, while those directly asserted for this concept are checked for consistency with the rest and used to distinguish this concept from other cutaneous melanomas. The role relationships can also be used, by human users or computer programs, to explore related information coded to the same or related molecular, pathologic or other features.

**Table 1:** NCI Thesaurus sample concept with new subtype concept

**A. Acral lentiginous melanoma (C4022)**(existing concept defined with both text and description logic role relationships)	
*Parent*: Cutaneous melanoma	
*Definition*: A form of melanoma occurring most often on the plantar, palmar, subungual, and periungual skin. It presents as a pigmented macular lesion with irregular borders. Morphologically, it consists of atypical spindled and dendritic melanocytes. The epidermis is often hyperplastic and there is pagetoid infiltration of the epidermis by anaplastic cells.	
*<Other synonyms, codes, etc.>*	
*Description logic role relationships*Inherits some values from more general concepts, adds five new ones.	
*Anatomy roles:*	
Disease_Has_Primary_Anatomic_Site	Skin of the extremity
Disease_Excludes_Primary_Anatomic_Site	Skin of the trunk
Disease_Has_Normal_Tissue_Origin	Skin tissue
*Pathology & finding roles:*	
Disease_Has_Normal_Cell_Origin	Melanocyte
Disease_Has_Abnormal_Cell	Dendritic melanoma cell
Disease_Has_Abnormal_Cell	Spindle melanoma cell
Disease_Has_Finding	Cutaneous involvement
Disease_Has_Finding	Macular lesion
*Molecular abnormality roles:*	
Disease_May_Have_Molecular_Abnormality	BRAF gene mutation
Disease_May_Have_Molecular_Abnormality	NF-2 tumor-suppressor gene inactivation

**B. Mucosal lentiginous melanoma (C48622)**(subtype concept added to support CAP protocol, with new text definition and role)	

*Parent*: Acral lentiginous melanoma	
*Definition*: An acral lentiginous melanoma affecting mucosal surfaces.	
*<Other synonyms, codes, etc.>*	
*Description logic role relationships*Inherits all values from parent concept above, add one to logically distinguish this concept.	
*Additional anatomy role:*	
Disease_Has_Normal_Tissue_Origin	Mucosa

The CAP protocol for cutaneous melanoma referenced a subtype of acral lentiginous melanoma not previously in NCI Thesaurus: *Mucosal Lentiginous Melanoma*. Adding this concept meant not only creating the term and code, but also adding a new role relationship (Disease_Has_Normal_Tissue_Origin = Mucosa) and definition text that clearly distinguish this concept from its parent (Table 1B).

In addition to its strong formal semantics, other significant considerations in choosing NCI Thesaurus included:

1. *Integration with other terminologies*: It is actively linked to the many diverse terminologies important to the cancer community through the NCI Metathesaurus, which connects 2,500,000 terms from over 50 terminologies. NCI Metathesaurus is built on top of a subset of the National Library of Medicine's Unified Medical Language System (UMLS) Metathesaurus [27,28].

2. *Integration with NCI's bioinformatics infrastructure*: Within NCI's caCORE architecture, NCI Thesaurus now provides direct referential semantics for all new object models and much of the common data element layer.

3. *Responsive expert curation*: Content is actively maintained by a large team of expert editors, who can often provide 24-hour turnaround to address simple needs and work interactively with NCI staff and partners to decide on the best approach to more complex ones. Updates can be used immediately for modeling and coding, and become available for public use through monthly server and data file releases.

4. *Open access*: All NCI Thesaurus content and services are freely available for worldwide use. Content is available through Web browsers, program APIs, and downloadable files in OWL, XML, and flat file formats.

## Results

We used the caCORE approach to develop UML models and semantic metadata for three CAP cancer checklists of common neoplasms – invasive breast carcinoma [29], invasive prostate carcinoma [30] and cutaneous melanoma [31].

A foundational objective of our work was to represent the scope and content of the existing CAP cancer checklists as faithfully as possible in our models. The requirements of the caBIG™ metadata modeling representation necessitated the addition of structural relationships and additional metadata that are not present within the CAP models. However, we neither added nor removed data elements or valid values from these representations.

### Terminology support

Throughout the entire modeling process, EVS staff worked closely with the project team to ensure that NCI Thesaurus terminology and semantics could accurately support both the overall information model and detailed coding requirements of the CAP protocols. While most needed terminology was already present, sometimes changes were needed, including the addition of new concepts, adding new term associations to existing concepts, and adding new areas of terminology representation.

Where a different term was being used for the same underlying meaning, the new CAP protocol term was added to an existing concept. For example, the term "macroscopic" was felt to have the same meaning as the NCI Thesaurus concept *Gross*, and was added to it. Similarly, the CAP protocol terms "unifocal" and "multifocal" were added to the existing *Unifocal Lesion *and *Multifocal Lesion *concepts, respectively.

In other cases, such as *Mucosal Lentiginous Melanoma*, *Lymphatic Invasion*, and *Venous Invasion*, a new concept was added. A whole new category of finding concepts was introduced to represent the details of cancer staging, most commonly done through the TNM (Tumor/Node/Metastasis) staging system. A set of general concepts was introduced to represent the various components and types of TNM staging, and then the specific staging components referenced in each of the three initial CAP protocols were added as separate concepts with logical hierarchies and text definitions in accordance with the current edition of the American Joint Commission on Cancer cancer staging manual. Pathologic staging terminology on cutaneous melanoma, breast cancer, and prostate cancer now covers the primary tumors (pT0, pT1, pT2, pT3, pT4, pTis, and pTX stage finding terms), regional lymph nodes involvement (pN0, pN1, pN2, pN3, and pNX stage finding terms), and distant metastasis (pM0, pM1, pMX stage finding terms).

### CAP cancer checklists information models

#### Uncovering and naming the high level classes

We first surveyed 15 of the 45 existing checklists in detail, created draft UML models to uncover commonalities, and then inspected remaining checklists to ensure generalization of these commonalities. We identified key general objects that intersected all checklists: SynopticSurgicalPathologyReport, SurgicalPathologySpecimen, Neoplasm, and CancerTNMFinding. We then incorporated additional objects to express complex datatypes and relationships, such as SurgicalMargins, ThreeDimensionalSize, NeoplasmHistologicType, and HistopathologicGrade.

Next, we identified data elements belonging to each high level object and assigned them as attributes of the class. For example, the histologic type of a neoplasm has a name such as "superficial spreading melanoma". Therefore the class NeoplasmHistologicType is given an attribute of 'name'. In parallel, we worked closely with the EVS terminologists to ensure that assigned names of classes and attributes were semantically unambiguous. When no existing NCI Thesaurus concept was identified, new vocabulary concepts were added. Alternatively, when concepts were deemed inappropriate for entry into the NCI Thesaurus, the concept was post-coordinated in the metadata using the object class or property and its qualifiers for data element concepts, or multiple value meaning codes for valid values. For example, the attribute Invasion Depth of CutaneousMelanoma was not added to the Thesaurus because its meaning was thought to be conveyed by the combination of two existing NCIT concepts. Thus, this attribute was annotated with "depth" (NCI C25333) as the *property *because it was considered to be the characteristic being defined, and "tumor cell invasion" (NCI C20625) as the *property qualifier *because it was considered to be a modifier of "depth". The result of this step in the process was to identify and name a general set of classes and attributes from which all specific CAP checklists could be developed.

#### Creating basic UML structure

**Figure 3:**
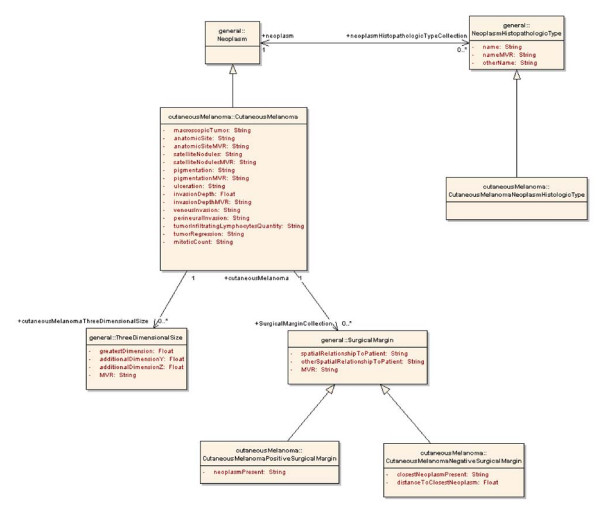
**Fragment of UML information model for melanoma**. Fragment of the CAP checklist UML class model showing association and generalization relationships of CutaneousMelanoma.

High-level UML classes were then joined by links representing the logical relationships between classes. Associative relationships were assigned directionality and cardinality. Inheritance relationships were used to extend the general classes as each specific CAP cancer checklist was developed. For example, CutaneousMelanomaNegativeSurgicalMargin expresses the surgical margin findings specific to negative margins in cases of cutaneous melanoma It is a subclass of the more general SurgicalMargin, and thus inherits all of the attributes of SurgicalMargin. CutaneousMelanomaNegativeSurgicalMargin also has additional attributes that are specific to negative margins. A small fragment of the total UML model is shown in Figure 3.

An important aspect of this phase of the modeling effort was to determine where generalization relationships should be structurally aligned with NCI Thesaurus. Semantic interoperability requires unambiguous semantics. One difficulty in utilizing a multi-structure modeling environment is that apparent conflicts may arise when the structures conflict. This could potentially produce significant ambiguity.

**Figure 4:**
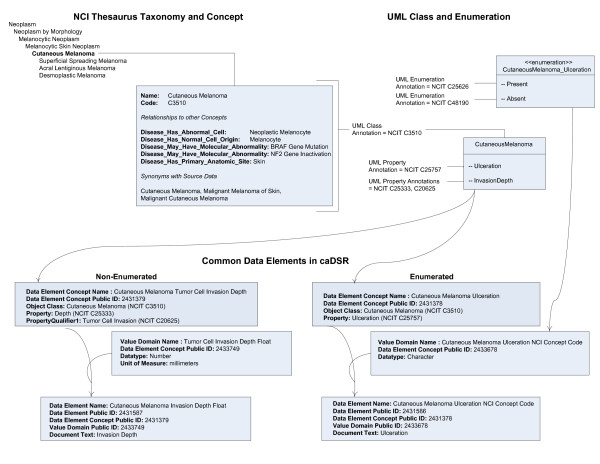
**Detailed view of structural relationships among information models, ontology, and semantic metadata**. CDEs are derived from ontology-annotated UML models. The figure depicts a single class with two attributes generating (a) CDE with a non-enumerated value domain, and (b) CDE with an enumerated value domain. A single NCI Thesaurus annotation is shown for the concept Cutaneous Melanoma. The relationship of the ISO/IEC metadata to UML model and NCI Thesaurus is also shown.

For example, Figure 4 shows an example of a single UML class (CutaneousMelanoma) with two attributes, and depicts how two separate CDEs are created. The UML class CutaneousMelanoma is shown in the larger UML model fragment (Figure 3) to be a subclass of the abstract UML class Neoplasm. Imagine that a user wishes to aggregate data from multiple sources about the depth of invasion of cutaneous melanomas. Since all classes are annotated with NCI Thesaurus concepts, it would be possible to use the caDSR to identify all classes of CutaneousMelanoma for which depth of invasion was an attribute, regardless of the actual names of these classes and attributes. Additionally, it would be possible to use the NCI Thesaurus to find all children of CutaneousMelanoma and then search the caDSR for classes related to these more specific diagnoses. Data on invasion depth from a class annotated as Superficial Spreading Melanoma, and data from a class annotated as Desmoplastic Melanoma could be aggregated with data from a class annotated as CutaneousMelanoma because both Superficial Spreading Melanoma and Desmoplastic Melanoma are children of CutaneousMelanoma in the NCI Thesaurus (Figure 4).

Now imagine that a particular UML model has a class annotated as Desmoplastic Melanoma, and that in this model it is a parent of a class annotated as CutaneousMelanoma. In this case there exists a relationship in the information model, which directly conflicts with the knowledge in NCI Thesaurus. If we attempt to aggregate all Desmoplastic Melanomas across multiple systems using the NCI Thesaurus taxonomy, we might falsely include data from CutaneousMelanomas that are not specifically Desmoplastic Melanomas. Synchrony between the UML and NCI structures are required to permit procedures based on valid inferences.

A basic principle we used was that when UML class and attribute names (object classes and properties in the metadata) were pre-coordinated in the vocabulary; we attempted to maintain structural synchrony with the NCI Thesaurus taxonomic tree. For example, in the case of cancer stage findings, a new branch of the NCI Thesaurus was developed to model these concepts, and the UML model uses object classes and properties that are annotated with single vocabulary tokens for object class and property, and no qualifiers. Thus, the annotations of the UML class tree for cancer stage findings are all structurally consistent with the NCI Thesaurus.

On the other hand, when UML class and attribute concepts (object classes and properties in the metadata) were post-coordinated in the metadata; it was not necessary or even possible to maintain structural synchrony with NCI Thesaurus. For example, the annotation of the UML class SurgicalPathologySpecimen is postcoordinated as Surgical Pathology (C16958) and Specimen (C19157). The subclass BreastSurgicalPathologySpecimen is also postcoordinated as Breast (C12971), Surgical Pathology (C16958) and Specimen (C19157). The relationship between BreastSurgicalPathologySpecimen and SurgicalPathologySpecimen exists only in the metadata and has no equivalent relationship in the NCI Thesaurus. The semantics of this metadata are constrained by the ISO/IEC 11179 specification and its implementation (see description in Methods section): one can determine from the metadata which part of the BreastSurgicalPathologySpecimen is the class and which is the attribute.

#### Representing specific classes by extension

Once the basic structure and general classes were established – we modeled three CAP checklists in their entirety. As described above, general classes were extended for each CAP checklist we modeled. For example, CutaneousMelanomaNegativeSurgicalMargin inherits three attributes from the general class SurgicalMargin, and extends this class with two additional attributes (Figure 3). Three CDEs will be created for Surgical Margin, and five will be created for CutaneousMelanomaNegativeSurgicalMargin.

Construction of classes and attributes sometimes required defining the datatype, which is not specified in the CAP protocol. When making these decisions, we attempted to balance potential uses. For example, we use the integer datatype for all Gleason Patterns and scores in order to facilitate calculations. An alternative method would be to use an enumeration of vocabulary tokens – each one representing a specific Gleason Pattern. These enumerations already exist in the NCI Thesaurus. Although the latter representation might be preferred for purposes of inference and information extraction, the need to easily compare numerical values and compute statistics such as mean and standard deviation were considered more important. An additional data element sharing the same DEC but differing in value domain could be constructed later if it was determined that the enumerated type was also necessary. The two CDEs resulting from this process would share the same DEC, indicating that the meaning of the data is identical but that the representations differ (concept code versus integer).

#### Packages

Classes were grouped into packages to define the natural aggregations and corresponding namespaces that arise from the paper standard. For example, all classes that are relevant to the CAP checklist for cutaneous melanoma are included in a single package. Packages become caDSR classification schemes during the UML Loading process.

#### Enumeration of valid values

In parallel to the creation of data elements, UML enumerations were used to express valid values for each of these data elements. Although no mechanism currently exists within caCORE for automating loading of these enumerations into caDSR, the use of UML enumerations greatly eased the metadata development process for the model developers, because it permitted us to maintain one set of artefacts for all of our metadata. The association between a value and an NCI Thesaurus concept further refines the semantics of data collected and helps to ensure its interoperability.

During the modeling process, we noted that in some cases attributes inherited from a common superclass to two subclasses should both inherit the same value domain. But in other cases, attributes inherited by two subclasses from a common superclass may require different value domains. In the first case, we consistently used the same value domain for both superclass and subclass during the post-load curation (see below), but in the second case we used distinct value domains.

We also found that some value domains could refer to entire sections of the NCI Thesaurus. In these cases, we created value domains by reference during post-load curation, such as the value domain for Metastasis Anatomic Site (value domain public ID 2433552).

In many cases, permissible values within a value domain represented reasons that particular values were absent, for example because the attribute was not identified or because the attribute could not be evaluated in this context. In these cases, we segregated 'missing value reasons' from the value domain proper, creating a separate attribute named <attribute-name>MVR, such as pigmentationMVR (Figure 3).

Value domains were created by transforming the Semantic Connector Excel reports, which contained valid values, into an alternate Excel format for loading into caDSR. Names in the value domains applied a convention to match them to the appropriate attribute. Further development of caCORE SDK will enable more automated methods for transformation of UML stereotypes into value domains.

### UML loading and post-load metadata curation

Annotated UML models were loaded into caDSR using the UML Loader, and value domains were loaded using the Excel Loader. The process for mapping and conversion of UML to ISO/IEC 11179 specified metadata in caDSR has been previously described [21,32].

Additional metadata curation steps were performed using the caDSR Curation Tool, including (1) revision of long names, (2) attachment of value domains to the corresponding data elements, (3) addition of data element derivation rules and form display text, and (4) inclusion of hyperlinks to the original documentation.

Once completed, the 174 CDEs were released and made publicly available. They may be inspected and downloaded in XML or to Excel using the caDSR Browser [33] or accessed programmatically at using the caCORE API [34].

## Discussion

We used the caBIG™ methodology to develop UML models with comprehensive ontology annotations and corresponding semantic metadata for a complex paper based clinical reporting standard. The process was labor-intensive and required significant cross-disciplinary collaboration between information modeling experts, terminologists, metadata curators, and domain experts. But recent advances in tool development should streamline some of the most time-consuming processes that we encountered. Establishment of appropriate local practices and expertise in model development and metadata curation required a significant initial investment. But now that practices and expertise have been established in our environment, further work can evolve more rapidly.

The process of implementing the CAP cancer checklists uncovered a number of issues that will directly affect future processes and representations.

### Implementation of value domains

One difficulty we had during modeling was the creation of value domains, for which there was not yet UML Loader support. The creation of these value lists was a post-curation activity, and depending on the number of enumerated attributes and the number of enumerations in each, it was another potentially laborious process. The caCORE 3.1 version of the SIW and UML Loader will be enhanced to allow the value domain information to be passed through the same semantic annotation process as the class and attributes. This feature will require that the model describes both a reference value domain in which the values are linked to a parent concept in the NCI Thesaurus, with all children as permissible values, as well as the ability to explicitly enumerate the values linked to NCI Thesaurus concepts in the permissible value list. The new process will support the annotation of the permissible values with NCI Thesaurus concepts and the ability to use and create a hierarchy of values reflective of the hierarchy in the source vocabulary from within the UML Model, as opposed to creating the annotation and hierarchy of values in the post-curation activity.

Another requirement we uncovered was the need to capture specific document text for permissible values that must be present on the CAP checklist, exactly as prescribed by the protocol authors. The need for descriptive text for permissible values is being addressed in future caDSR releases by elevating the value meaning to an administered item in caDSR, for which curators can then specify alternate names, definitions and document text similar to CDEs. In this case, the descriptive permissible value for use on the CAP checklist can be entered as a reference document of type "Preferred Question Text".

A third new feature that will be added to caCORE 3.1 is that the attribute can optionally point to an existing value domain, again reducing post curation workload and streamlining the creation of CDEs.

### Expressivity of semantic annotation

In a small number of cases, the method for semantic annotation was not sufficiently expressive to convey the meaning of a particular datum. For example, the class InvasiveBreastCarcinoma had an attribute named venouslymphaticInvasion that could take values absent, present, or indeterminate. The attribute indicates whether there is tumor invasion of small veins and/or tumor invasion of lymphatics. Often it is difficult or impossible to determine whether a particular vessel is a venous channel or a lymphatic channel, and so a distinction is not made. This meaning was difficult to express because the ordinal nature of concept annotation of properties required one and only one property modified by one or more qualifiers. In this case there was no way to convey the equivalent status of the two concepts *venous invasion *and *lymphatic invasio*n paired by the conjunctive *and/or*. Although a newly developed model might choose to represent this as two distinct attributes, existing data models such as those based on the CAP cancer checklists do not capture data at this level of granularity and therefore semantic annotation must provide a method to express such constructions.

In most cases the strictly ordinal, non-nested relationship of qualifiers to a property was sufficient to unambiguously express the meaning when semantic meaning was post-coordinated in the metadata. For example, invasionDepth could be expressed using two concepts: property = *depth *and qualifier1 = *tumor cell invasion*. But in other cases, conceptual relationships inherent to a property were poorly expressed without some way to nest these qualifiers. For example for the attribute incidentalProstaticTissueInvolvementPercentage – we used four concepts – three qualifiers for (1) *incidental*, (2) *prostatic tissue*, and (3) *involvement*, and the property *percentage*. The annotation set can be represented as the list:

(incidental (prostatic tissue (involvement (percentage))))

But the simple ordinal nature of the qualifiers did not allow us to express the meaning behind the intended more complex linguistic construction, which is best expressed as the list:

(incidental (prostatic tissue (involvement)) percentage)

leaving the interpreter unable to determine whether it is the 'prostatic tissue' or the 'prostatic tissue involvement' that is incidental.

In the next generation of caDSR, an enhanced concept derivation rule will allow users to specify Boolean operators between groups of concepts. Instead of strictly ordinal positions, the qualifiers will be grouped as 'levels' or 'ordinal groups'. Between each group, a Boolean expression can be entered to indicate explicitly the relationship between to the term the group modifies. Using the same example from above, the attribute incidentalProstaticTissueInvolvementPercentage – would be represented using the same concepts, but adding the Boolean expression "and" between qualifier group (2) and group (3):

(incidental (prostatic tissue AND involvement) percentage)

The annotation set can still be represented as the list, with the optional inclusion of the Boolean operator between ordinal groups.

### Need for more formal semantic annotation guidelines

An underlying assumption of the semantic metadata annotation process is that, given a particular UML entity, there is one most valid conceptual representation. For example, the UML class *protein *should always be annotated with NCI Thesaurus concept C17021, and the UML attribute *identifier *should always be annotated with NCI Thesaurus concept C25364. If the annotation of such semantically equivalent entities is highly reproducible, we may be able to aggregate across datasets, and obtain valid results, emergent from separate, partially overlapping models. But if semantic annotation is not reproducible, then semantically equivalent data may be annotated with different concepts or non-equivalent data may be annotated with identical concepts. In either case, aggregation is likely to produce erroneous results.

For the authors of this manuscript, the distinction between qualifiers and properties has proved to be among the most difficult aspects of establishing consistent human annotations. Determining which term should be used as the property and which terms should be qualifiers often seems somewhat arbitrary. These human decisions could be made more consistent by establishing more formal guidelines for making these choices, such as those developed for other human annotation tasks [35]. To date, there have been no systematic studies of the inter-rater or intra-rater reliability of semantic annotation using this methodology. Further work is needed to establish the reproducibility of metadata annotation.

### Asynchronous structures in the multi-tiered models

The three-tier caCORE modeling environment is designed to maintain core canonical knowledge in the terminology/ontology layer, while contextualizing semantic meaning in the structured metadata. An important issue we recognized with this approach is that semantics expressed in the information model and metadata may not be expressed in the ontology, or may contradict semantics expressed in the ontology. The description logic, UML representations, and ISO/IEC 11179 compliant metadata represent three different graph structures that partially overlap and may be asynchronous.

Where the three layers are not properly synchronized, it may not be possible to make valid inferences about the contextualized meanings represented in the information models and structured metadata. Neither UML nor ISO 11179 have formal model-theoretic semantics. Because we do not tightly constrain the information models to the ontology, it is likely that we will be limited in the kinds of inference that will be possible. Even the potential to use the NCI taxonomies for purposes of aggregation across models (using virtual superclasses derived from the ontology) may be limited if the information models express hierarchical relationships that directly contradict the taxonomies.

The potential for asynchrony is not all bad. In fact, it is often necessary to express semantics which contradict the formal, canonical representation. Researchers must be free to pose and model hypotheses that directly contradict accepted beliefs. In fact, the ability of these contextualized models to act as a source for new ontology concepts and relationships is significant. As scientific knowledge evolves, the semantics expressed in these more informal knowledge representations could provide a conduit for reverse engineering, driving ontology development from the bottom up as well as from the top down.

### Advantages of the caCORE approach

Despite the limitations described above, we found that the caBIG™ methods and the results of these methods offer very significant advantages in developing interoperable enterprise informatics systems:

#### • Common information model

We have already begun to reap the benefits of a single, reusable representation of the CAP cancer checklists in our environment. The potential to connect Cancer Text Information Extraction System (caTIES), Clinical Annotation Engine (CAE) and our CoPathPlus clinical archives through this representation offers opportunities for supporting tissue-based research that would be simply impossible without a shared information model.

#### • Binding to controlled vocabulary

The binding to controlled vocabulary provides the opportunity to reference human readable definitions at the time of data entry as well as data receipt. We anticipate that this will enhance the accuracy of data entry as well as later interpretation.

#### • Reuse across multiple systems

The implementation neutrality and accessibility of the semantic metadata has enabled us to reuse the same metadata for multiple purposes including populating user interfaces, mapping from legacy data, and extracting information from text.

#### • Potential for inference using the NCI Thesaurus

One potential advantage of the methodology is to use the strong formal semantics of the NCI Thesaurus to manipulate data, both within a single system (e.g. caTIES) and across systems (e.g. caGrid). The ability to use NCI Thesaurus to make inferences regarding the information models will depend on the fidelity of the semantic annotations and ontology associated with this metadata.

### Uses of the CAP cancer checklists model and metadata

The CAP checklist models and metadata are intended to become a caBIG™ data standard that can serve multiple purposes. There are four applications currently working on incorporating the CAP metadata – two caBIG™ products, one vendor application, and one research educational application. These applications demonstrate the diversity of uses that can be supported by such a semantically annotated electronic data standard.

#### CAE

CAE is an N-tier Web application for entry and retrieval of clinical data about cancer patients and the biospecimens collected from them. CAE allows cancer centers to integrate data from a variety of clinical systems and supplement that data with manual annotations as necessary.

CAE uses the CAP cancer checklist models in two ways. First, it directly reuses classes derived from the CAP cancer checklist UML model. Consequently, UML loading of the CAE model resulted in assignment of the existing CAP CDE to each matching class-attribute pair in the CAE object model. As additional applications use the CAP cancer checklist CDEs, data can be aggregated from multiple sources.

**Figure 5:**
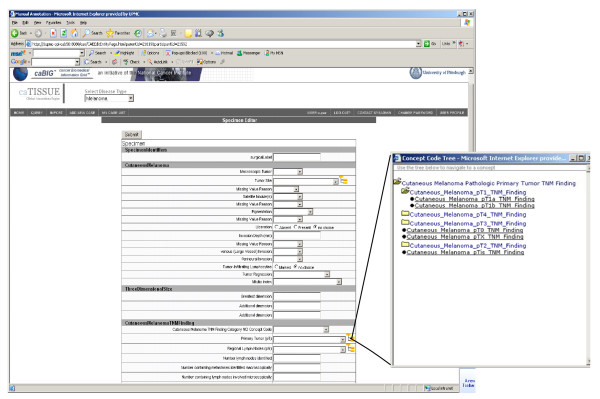
**CAE manual annotation interface**. The generic CAE user interface utilizes CAP cancer checklist metadata to populate screens, and drop down boxes, and reuses objects from the CAP cancer checklist UML model as part of the CAE information model. In this figure, value domains by reference are indicated with the tree icon. The user can browse and select any value below the root node designated in the metadata.

Second, CAE user interfaces for LIS import and manual annotation are directly derived from the metadata stored in caDSR (Figure 5). Labels utilize caDSR data element long names or document text. Drop-down menus are populated with valid values from the value domain for that CDE. In cases where the value domain is defined by reference to a single EVS vocabulary node, the manual annotation interface opens an NCI Thesaurus tree browser to the node for use in selecting a child concept.

#### caTIES

caTIES is a caBIG™ Silver compatible system for concept-based indexing and retrieval of surgical pathology reports. The system takes free-text pathology reports, breaks them into sections, maps free-text data within these sections to NCI Metathesaurus concepts, and stores these concepts and conceptual relationships. Researchers may query the indexed datastore to retrieve documents and order tissue through honest brokers.

A key development goal for caTIES is to evolve towards a true information extraction system. caTIES is already able to recognize key features such as the diagnosis, procedure, and organ, but it does not extract any of the more finely granular data such as whether or not lymph nodes are involved or the depth of invasion of the lesion. An important problem in extracting these variables is knowing which variables should be sought given a particular context. Attempts to extract all variables are resource intensive and can result in false positive errors. The CAP cancer checklist model metadata facilitates the development of these capabilities in several ways.

First, the classification schemes created by the CAP checklists provide a set of templates that caTIES can use to categorize relevant information once the necessary template is known. For example, if it can be determined that the particular case is a wide excision of cutaneous melanoma as opposed to a radical prostatectomy for prostate carcinoma, then the set of data elements that may be sought can be restricted to those within the classification scheme for this diagnosis-procedure pairing.

Second, NCI Thesaurus annotations provide a method for determining the diagnosis-procedure pairing. Each classification scheme is associated with one UML class that encapsulates information regarding the tumor and one class that encapsulates information regarding the specimen. caTIES will attempt to use the NCI Thesaurus concepts that describe these classes and attributes to determine whether a particular template is relevant. For example, caTIES can already identify superficial spreading melanoma as a diagnosis. Therefore, by inference using the NCI disease classification hierarchy, caTIES could determine that the cutaneous melanoma classification scheme is the template that should be used for extraction.

The creation of the CAP cancer checklist models tied to the NCI Thesaurus is the critical first step in developing ontology-driven information extraction methods for caTIES.

#### Cerner CoPathPlus caBIG™ Data Extractor

Many existing LIS capture data that are compatible with the CAP protocols but use proprietary relational designs. An important aspect of this work is to make these caBIG™ standards freely available and to encourage vendors to annotate existing data with them. CoPathPlus, a Cerner anatomic pathology laboratory (APL)-LIS product, has created the caBIG™ Data Extractor as a tool for exporting data from their proprietary database and providing it to caBIG™ compliant systems. The caBIG™ Data Extractor is available for CoPath Plus v2.5. It is currently deployed at the University of Pittsburgh Medical Center, where it is being used to populate caBIG™ systems such as caTIES. The current tool exports coded data from synoptic reports that are annotated with the SNOMED CAP vocabulary. We are currently working with Cerner DHTI to map the CAP cancer checklist CDEs to the CoPathPlus representation of the CAP protocols, so that future versions of the Extractor will provide caDSR public IDs with each coded field. Other caBIG™ systems will therefore be able to directly import CoPathPlus coded synoptic data into data structures based on the CAP cancer checklist models.

#### ReportTutor

ReportTutor is an extension to our work on intelligent tutoring systems for visual diagnosis [36]. ReportTutor combines a virtual microscope and a natural language interface to allow students to visually inspect a virtual slide as they type a diagnostic report on the case. The system monitors actions in the virtual microscope interface as well as text created by the student in the reporting interface. It provides feedback about the correctness, completeness, and style of the report. ReportTutor uses MMTx with a custom data-source created with the NCI Metathesaurus. A separate ontology of cancer specific concepts is used to structure the domain knowledge needed for evaluation of the student's input, including co-reference resolution. ReportTutor uses the data elements from the CAP cancer checklists for melanoma to structure the interaction with the student. We are currently working on extending the system by creating a generic harness that will load the caDSR metadata for each classification scheme as a new module for creating ReportTutor cases.

### Future work on the CAP cancer checklists models

The three models we have developed will form the foundation for future work to develop all 45 CAP checklists as part of one common information model. As work advances, we hope to move this model towards acceptance as a caBIG™ standard, following a well-described process devised to promote data standard development [37]. For the CAP cancer checklists, this process must involve a thorough domain review to establish the accuracy of the representation when compared to the intent of the authors of original protocols.

## Conclusion

The CAP cancer checklists model provides a case study of early data standard implementation using the caBIG™ methodology. The models and associated metadata are publicly available, and are currently being used within a variety of applications. The results of our work identify some limitations of the caBIG™ methods, which will inform future versions of processes and technologies. However, our results also highlight the effectiveness and potential for semantic integration of biomedical informatics systems using a multi-tier modeling approach.

## Competing interests

The author(s) declare that they have no competing interests.

## Authors' contributions

JT, RC, SM and RSC carried out the UML modeling. LW, NS and RSC performed the semantic annotation. LW and NS developed required vocabulary in the NCI Thesaurus. RC, DBW, GAK and RSC participated in the overall information model and metadata design. All authors contributed in drafting the manuscript, and read and approved the final document.

## Pre-publication history

The pre-publication history for this paper can be accessed here:


